# Non-Mass Lesions on Automated Breast Ultrasound: An Exploratory Analysis of Imaging Features Associated with Malignancy and Differentiation from Transient Non-Mass ABUS Findings

**DOI:** 10.3390/diagnostics16142186

**Published:** 2026-07-14

**Authors:** Gahyeon Kim, Jieun Kim, Hyun Kyung Jung, Suk Jung Kim

**Affiliations:** 1 Department of Radiology, Inje University Haeundae Paik Hospital, Busan 48108, Republic of Korea; h80850@paik.ac.kr (G.K.);; 2 Department of Radiology, Miraero Hospital, Busan 46544, Republic of Korea

**Keywords:** non-mass lesions, transient non-mass ABUS findings, automated breast ultrasound, coronal plane, BI-RADS

## Abstract

**Objectives**: This study aimed to investigate imaging features associated with malignancy and to differentiate true non-mass lesions from transient non-mass ABUS findings on automated breast ultrasound (ABUS). **Methods**: This retrospective study included 133 women with non-mass lesions, each contributing a single lesion assessed as BI-RADS categories 0, 4, or 5 on ABUS between January 2020 and October 2022. We analyzed multiple ABUS imaging features, including background echotexture, distribution, microcalcification-like echogenic foci, posterior shadowing, architectural distortion on axial and coronal views, and volume change. These findings were statistically compared according to pathologic results for malignancy and ultrasonographic correlation for true versus transient non-mass ABUS findings. **Results**: Among the 133 lesions, 15 (11.3%) were malignant and 65 (48.9%) were transient non-mass ABUS findings. Segmental distribution (odds ratio [OR] = 40.42; 95% confidence interval [CI], 4.45–367.27; *p* = 0.001) and coronal architectural distortion (OR = 53.59; 95% CI, 6.26–458.90; *p* < 0.001) showed exploratory associations with malignancy. A non-heterogeneous background echotexture (OR = 0.07; 95% CI, 0.02–0.33; *p* = 0.001), lack of posterior shadowing (OR = 0.14; 95% CI, 0.03–0.60; *p* = 0.008), and volume change (OR = 15.79; 95% CI, 1.47–170.12; *p* = 0.023) were associated with true lesions. **Conclusions**: Segmental distribution and coronal architectural distortion were associated with malignancy on ABUS in this exploratory study. Lack of posterior shadowing and the presence of volume change were associated with true lesions, while heterogeneous background echotexture was associated with transient non-mass ABUS findings. These ABUS findings may contribute to the characterization of non-mass lesions but, given the small number of malignant lesions, require cautious interpretation and validation in larger studies.

## 1. Introduction

The early detection of breast cancer is the primary goal of breast screening [1]. Breast ultrasonography (US) is widely used to evaluate breast lesions. Handheld breast ultrasound (HHUS) is conventionally used but has limitations such as operator dependency and reproducibility issues [2]. To address these challenges, automated breast ultrasonography (ABUS) was introduced to provide three-dimensional volumetric imaging with reconstructed coronal and sagittal planes [3,4]. Nonetheless, ABUS has its own issues, including frequent transient non-mass ABUS findings caused by heterogeneous background echotexture and coronal slab artifacts [5]. Additionally, suboptimal image quality due to various artifacts such as reverberation, skipping, motion, and section thickness artifacts can impede accurate diagnosis [2,6,7]. These limitations may be particularly relevant when interpreting non-mass findings, because artifacts or heterogeneous background parenchyma can be difficult to distinguish from true non-mass lesions.

Several studies have compared ABUS and HHUS in terms of diagnostic performance, with no significant differences in interobserver variability and diagnostic accuracy [8,9]. Studies based on the fifth edition of the Breast Imaging Reporting and Data System (BI-RADS) by the American College of Radiology (ACR) reported good interobserver agreement for suspicious masses, and ABUS sometimes provided important additional information [10,11]. ABUS tended to assign lower BI-RADS categories than HHUS for small malignant breast masses (≤1 cm) while maintaining suspicion within category 4A or higher, and these characteristics have contributed to its acceptance as an effective screening tool that reduces false positives while improving cancer detection [12]. However, these studies largely focused on diagnostic performance or BI-RADS assessment of mass lesions rather than non-mass lesions. Therefore, findings from previous ABUS studies may not be fully applicable to ABUS-detected non-mass lesions, which have different imaging characteristics and diagnostic challenges.

According to the ACR BI-RADS v2025 update, a non-mass lesion is newly defined as a discrete three-dimensional finding on US that lacks the distinct margins and definable shape of a mass and is recognized by disruption of the background parenchymal echotexture [13]. Non-mass lesions encompass various pathologies, from benign to malignant [14,15]. Previous attempts to stratify the malignancy risk of non-mass lesions have been based mainly on HHUS. Park et al. developed a classification system to stratify the cancer risk of non-mass lesions detected using HHUS [14]. They found that segmental distribution, associated calcifications, abnormal ductal changes, and posterior shadowing were associated with malignant non-mass lesions. However, HHUS-based descriptors do not fully account for ABUS-specific features, such as coronal-plane architectural distortion and artifacts related to automated acquisition. A recent review article has shown that ABUS is helpful for evaluating non-mass lesions that exhibit extensive and parallel orientation [16]. Particularly on the coronal view, ABUS can detect subtle architectural distortions that are easily missed by HHUS. Zhang et al. showed that ABUS was superior to mammography in determining malignant non-mass lesions and that the coronal plane of ABUS had a better detection rate for architectural distortion than mammography and HHUS [17]. However, the ABUS-specific imaging features associated with malignancy in non-mass lesions remain less well characterized.

Beyond malignancy assessment, another challenge in ABUS interpretation is determining whether an ABUS-detected non-mass finding represents a true non-mass lesion rather than a transient non-mass ABUS finding. Such transient findings may arise from heterogeneous background echotexture, artifacts, or compression-related factors. Most previous studies have focused on benign-versus-malignant differentiation, and imaging features that may help distinguish true non-mass lesions from transient non-mass ABUS findings remain unclear. Accordingly, a practical diagnostic gap remains in the interpretation of ABUS-detected non-mass findings, encompassing both assessment of malignant potential and differentiation of true non-mass lesions from transient non-mass ABUS findings. Therefore, this exploratory study aimed to investigate ABUS imaging features associated with malignancy among true non-mass lesions and to explore features that may help differentiate true non-mass lesions from transient non-mass ABUS findings.

## 2. Materials and Methods

### 2.1. Study Population

**Figure 1:**
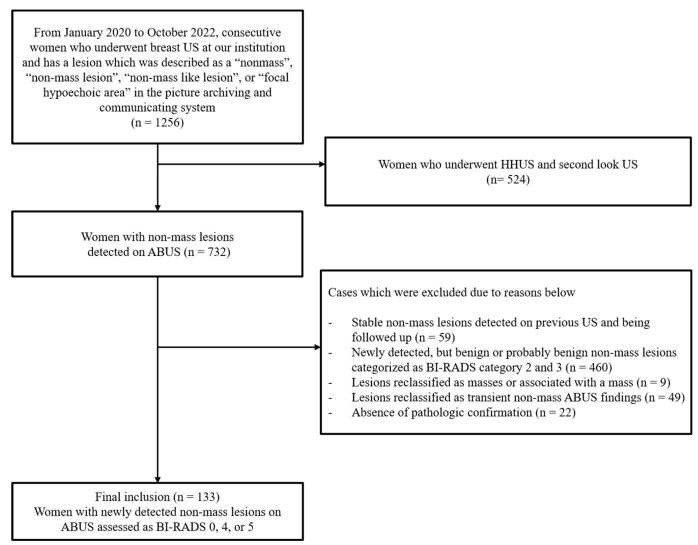
Flowchart of patient enrollment process.

This study was approved by our institutional review board (IRB No. 2023-08-010-001), and the requirement for informed consent was waived due to the study’s retrospective design. From January 2020 to October 2022, a total of 1256 consecutive women underwent breast US at our institution, presenting with lesions described as “nonmass,” “non-mass lesion,” “non-mass like lesion,” or “focal hypoechoic area” in our institution’s picture archiving and communication system. The included lesions were considered compatible with the ACR BI-RADS v2025 definition of a non-mass lesion, which describes a discrete three-dimensional finding that lacks distinct margination and a specific shape of a mass and is recognized by disruption of the background parenchymal echotexture [13]. After excluding women who underwent HHUS and second-look US (*n* = 524) during the retrospective review, non-mass lesions were detected on ABUS in 732 women. Among these patients, 599 were excluded based on the following criteria: stable non-mass lesions detected on previous US and being followed up (*n* = 59), newly detected but benign or probably benign non-mass lesions categorized as BI-RADS categories 2 and 3 (*n* = 460), lesions reclassified as definite masses or associated with a mass (*n* = 9), lesions reclassified as definite transient non-mass ABUS findings (*n* = 49), and absence of pathologic confirmation (*n* = 22). The term “transient non-mass ABUS finding” refers to a non-reproducible ABUS finding without a sonographic correlate on second-look or follow-up US that is subsequently attributed to normal breast parenchyma or imaging artifacts, including compression artifacts, Cooper ligament shadowing, and normal parenchymal variation. Newly detected BI-RADS category 2 and 3 lesions on ABUS were excluded because these lesions are generally managed without histopathologic confirmation in routine clinical practice. Accordingly, this study focused on 133 women (mean age, 50.75 years; range, 39–62) with newly detected non-mass lesions assessed as BI-RADS categories 0, 4, or 5, which were considered clinically actionable and suitable for analysis of imaging features associated with malignancy and differentiation from transient non-mass ABUS findings (Figure 1). Each woman contributed a single target lesion to the analysis.

### 2.2. Image Examination and Interpretation

ABUS was performed by a well-trained technologist using an Invenia ABUS system with an automated 6–15 MHz, 15.3 cm wide-field view transducer (Reverse Curve™, Ultra-broadband Transducer, GE Healthcare, Sunnyvale, CA, USA). Whole breasts were scanned bilaterally in the anteroposterior, medial, and lateral orientations. Supplementary scans were performed if the basic three scans did not include all the relevant breast tissue. The patients were placed in the supine position with the ipsilateral arm raised and a sponge beneath the shoulders to spread the breast tissue. The scan depth was approximately 4–5 cm and encompassed the skin and chest wall muscles, including the pectoralis major and intercostal muscles. Standard vendor-provided post-processing and reconstruction were applied using the nipple position as a reference. The acquired volume data were automatically transferred from the ABUS scanner to a review workstation. Subsequently, the volume data were reconstructed in the axial, sagittal, and coronal planes at the ABUS workstation, maintaining a 0.5 mm slice interval.

**Figure 2:**
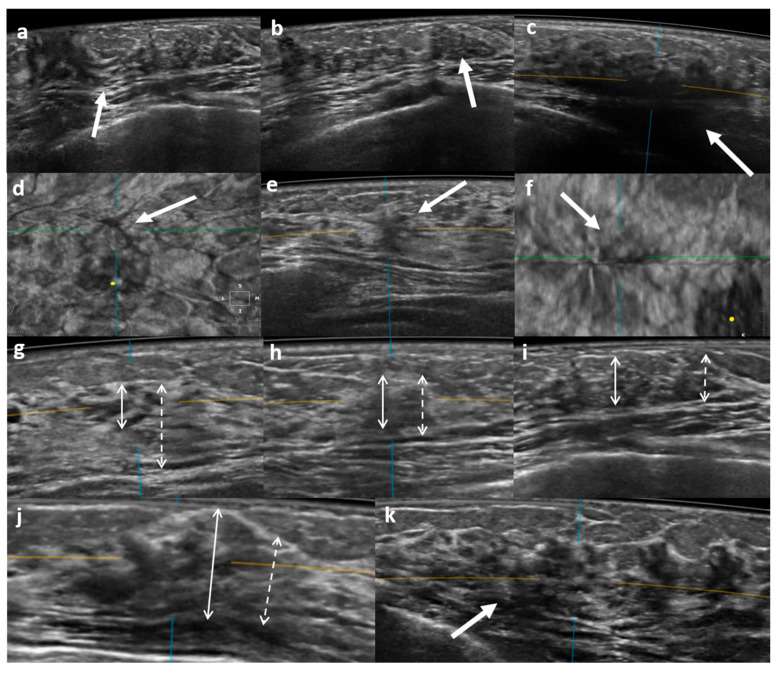
Representative imaging findings (arrows) of a non-mass lesion detected on automated breast ultrasonography (ABUS). (**a**) Segmental distribution, defined as a triangular or cone-shaped hypoechoic area with the apex pointing toward the nipple. (**b**) Microcalcification-like echogenic foci, defined as echogenic foci with or without posterior shadowing. (**c**) Posterior shadowing, defined as a distinct hypoechoic region posterior to the non-mass lesion. Architectural distortion on the (**d**) coronal and (**e**) axial plane, defined as distortion of the normal tissue architecture with radiating lines or focal retraction around the non-mass lesion. (**f**) Skip artifact, defined as a horizontal step-like line on the coronal plane caused by abrupt changes in transducer contact or compression during automated scanning. The degree of fibroglandular tissue (FGT) involvement (arrows) relative to the surrounding FGT (dotted arrows) was categorized as (**g**) <50%, (**h**) ≥50%, or (**i**) whole involvement. (**j**) Volume change, defined as a >10% increase in the extent of fibroglandular tissue (arrow) compared with the surrounding tissue (dotted arrow). (**k**) Heterogeneous background echotexture, defined as the presence of multiple regions of increased and decreased echogenicity within the breast parenchyma.

Initial interpretations of ABUS were performed by one of three dedicated breast radiologists (with 16, 14, and 4 years of experience) at the time of clinical diagnosis. The imaging findings and assessment categories of breast non-mass lesions on ABUS were evaluated according to the fifth edition of the BI-RADS US criteria, which was in clinical use during the study period. A specialized breast radiologist (4 years of experience) performed a retrospective analysis of various US findings of non-mass lesions without knowledge of the pathologic outcome or clinical information. Mammography and digital breast tomosynthesis (DBT) were not systematically reviewed or correlated with ABUS findings in this study. The ABUS image findings were analyzed based on the following parameters: (a) lesion size measured as the maximum diameter on the plane where the lesion was most conspicuous; (b) internal echogenicity dichotomized into isoechogenicity and hypoechogenicity; (c) segmental distribution defined as a triangular or cone-shaped hypoechoic area with the apex pointing to the nipple [18]. Segmental distribution was analyzed separately in this study based on prior studies demonstrating its higher association with malignancy [14]. Other BI-RADS distribution patterns (focal, linear, and regional) were grouped as non-segmental patterns for comparative analysis, both to maintain consistency with prior literature and to account for the limited number of cases in each individual category. (d) microcalcification-like echogenic foci as echogenic signals with or without associated shadowing; (e) posterior shadowing as a distinct darker region located posterior to the non-mass lesions; (f) ductal change; (g) cystic change; (h) architectural distortion as distortion of the normal tissue architecture with radiating lines or focal retraction, assessed on both coronal and axial planes; (i) skip artifact defined as a horizontal step-like line on the coronal plane caused by abrupt changes in transducer contact or compression during automated scanning [19]; (j) lesion location classified as periareola, periphery, or neither; (k) involvement degree of fibroglandular tissue (FGT) categorized as less than half, more than half, or whole involvement; (l) volume change was defined as a more than 10% increase in FGT compared with the surrounding tissue. This was assessed on the axial plane at the level where the lesion showed the greatest vertical extent. The lesion thickness was compared with adjacent normal FGT located approximately 1 cm from the lesion margin, selecting the side with preserved normal parenchyma. Measurements were obtained using picture archiving and communication system tools, and volume change was determined semi-quantitatively based on visual estimation as present or absent, rather than by automated volumetric measurement; and (m) background echotexture divided into homogeneous and heterogeneous. Homogeneous echotexture was defined as a uniform echogenic pattern, and heterogeneous echotexture was defined as mixed areas of increased and decreased echogenicity in the breast parenchyma [5] (Figure 2).

### 2.3. Reference Standards

All non-mass lesions were classified as true lesions versus transient non-mass ABUS findings. “True lesion” is not a standard BI-RADS term but was used as a study-specific operational category to denote ABUS findings with pathologic confirmation or a persistent sonographic correlate on second-look or follow-up imaging. True lesions were divided into benign and malignant groups. Non-mass lesions assessed as BI-RADS 4 or 5 on ABUS or second-look US underwent US-guided core needle biopsy. Surgical pathology was incorporated when available, and the final pathologic diagnosis was used as the reference standard for malignancy. Malignancies were defined as invasive ductal carcinoma, invasive lobular carcinoma, and ductal carcinoma in situ (DCIS). All non-malignant pathological outcomes, including high-risk lesions, were classified as benign for the analysis of imaging features associated with malignancy. When the initial BI-RADS category was 0 on ABUS, a second-look US was performed within 2 weeks. When second-look US was performed, non-mass lesions interpreted as final BI-RADS category 2 or 3 were categorized as benign. When second-look US was not performed, but follow-up ABUS or HHUS was performed, non-mass lesions that remained stable for at least 12 months were classified as benign.

Transient non-mass ABUS findings were also identified using second-look or follow-up US. Transient non-mass ABUS findings were defined as findings on ABUS without a corresponding lesion on second-look or follow-up US. This included cases that were assessed as final BI-RADS category 1 or 2 (but confirmed as fat, parenchyma, or definite shadowing by Cooper’s ligaments) on second-look US or cases that either disappeared or showed definite shadowing artifacts on follow-up US. Table 1 summarizes the reference standards.

### 2.4. Statistical Analysis

ABUS findings were compared between (1) benign vs. malignant lesions and (2) true lesions vs. transient non-mass ABUS findings. Regarding the comparison analysis between true and transient non-mass ABUS findings, true lesions included both benign and malignant pathologies. Intergroup comparisons of categorical variables were performed using Pearson’s chi-square or Fisher’s exact tests. Continuous variables (age and lesion size) were compared using an independent *t*-test or the Mann–Whitney U test. A *p*-value < 0.05 was considered statistically significant. Variables with *p* < 0.05 in univariable analyses were considered for inclusion in the multivariable logistic regression model. However, given the limited number of malignant cases, the number of variables entered into the model was intentionally restricted to reduce the risk of overfitting, and clinically relevant lesion-related variables were prioritized. Odds ratios (ORs) with 95% confidence intervals (CIs) were calculated. Data were analyzed using SPSS Statistics (version 25.0; IBM, Armonk, NY, USA).

## 3. Results

Of the 133 newly detected non-mass lesions on ABUS, 65 (48.9%) were categorized as transient non-mass ABUS findings, and 68 (51.1%) were categorized as true lesions, which were classified as benign (53 lesions, 39.8%) and malignant (15 lesions, 11.3%). Among the 53 benign non-mass lesions, 39 lesions (73.6%) were confirmed through core needle biopsy, including four cases of fibroadenomas, 32 cases of fibrocystic changes, and three cases of sclerosing adenosis. The remaining 14 lesions (26.4%) were classified as benign based on imaging findings: 12 lesions (22.6%) were classified as BI-RADS category 2 or 3 on second-look US examinations, and two lesions (3.8%) were classified as benign based on stability for at least 12 months on follow-up US. All 15 malignant lesions were pathologically confirmed and comprised eight invasive carcinomas (seven invasive ductal carcinomas and one invasive lobular carcinoma) and seven pure DCIS lesions. Surgical pathology was available for seven of the eight invasive carcinomas, all of which were Nottingham combined histologic grade 2; the remaining invasive ductal carcinoma, without available surgical pathology, was nuclear grade 2 on core needle biopsy. Associated DCIS components were identified in five of the seven invasive carcinomas with available surgical pathology, including four with an extensive nuclear grade 3 DCIS component. Among the seven pure DCIS lesions, one was nuclear grade 2 and six were nuclear grade 3. Pathologic microcalcifications were identified in 12 of 13 malignant lesions with available pathologic information on microcalcification status; this information was unavailable in the remaining two biopsy-only lesions. In a descriptive post hoc review, available mammograms of the malignant lesions were reviewed for potential mammographic correlates; no additional statistical analysis was performed. Mammograms were available for 14 of the 15 malignant lesions. A mammographic correlate was identified in 11 lesions, including microcalcifications in 9, architectural distortion in 3, focal asymmetry in 3, and a mass in 2, with some lesions showing overlapping findings. The remaining 3 lesions had no definite mammographic correlate.

Among the 65 transient non-mass ABUS findings, most were initially classified as BI-RADS category 0 on the initial ABUS. Three lesions were reclassified as transient non-mass ABUS findings after initially being categorized as category 4A on the initial ABUS.

Among the 68 true non-mass lesions, the clinical and ABUS findings of benign and malignant lesions are summarized in Table 2. Higher final BI-RADS category, segmental distribution, presence of microcalcification-like echogenic foci, presence of ductal change, architectural distortion on the coronal plane, and skip artifacts on the coronal plane were associated with malignancy, whereas less than half involvement of FGT was associated with benign results. There were no significant differences between benign and malignant lesions regarding patient age, lesion size, internal echogenicity, presence of posterior shadowing, cystic change, architectural distortion on the axial plane, location of the lesion, volume change, and background echotexture.

**Figure 3:**
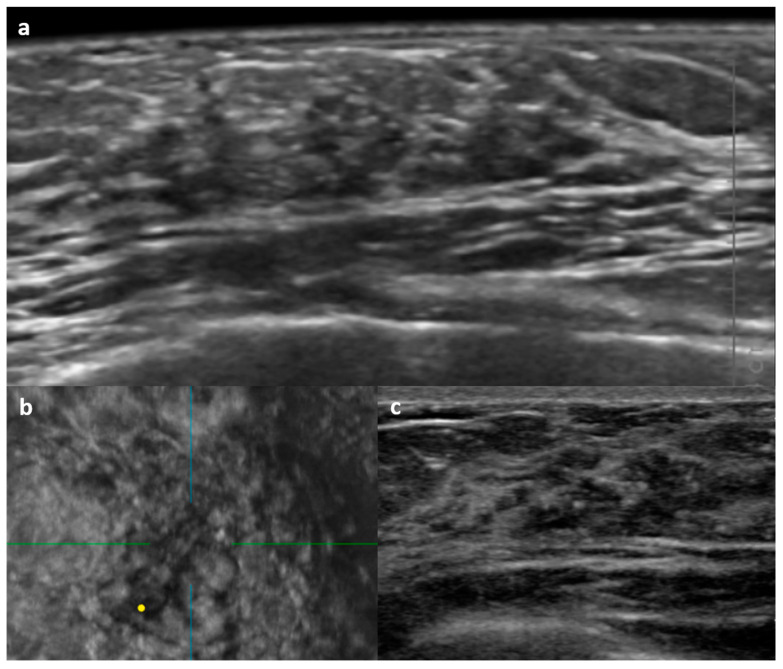
A 32-year-old woman diagnosed with ductal carcinoma in situ by core needle biopsy and subsequent surgery. (**a**) The axial plane of automated breast ultrasound (ABUS) shows a non-mass lesion with segmental distribution, microcalcification-like echogenic foci, volume change, and whole fibroglandular tissue (FGT) involvement in the upper outer quadrant of the left breast. (**b**) The coronal plane of ABUS shows segmental distribution and subtle architectural distortion. (**c**) Handheld breast ultrasound (HHUS) shows imaging findings similar to those on the axial plane of ABUS.

Among the 68 true non-mass lesions, multivariable analysis was performed to identify imaging features associated with malignancy (Table 3). Segmental distribution (OR = 40.42; 95% CI, 4.45–367.27; *p* = 0.001) and architectural distortion on the coronal view (OR = 53.59; 95% CI, 6.26–458.90; *p* < 0.001) showed exploratory associations with malignancy (Figure 3). When these two features were considered together, two of the 15 malignant lesions (13.3%) showed neither segmental distribution nor architectural distortion on the coronal view. Posterior shadowing was not significantly associated with malignancy (OR = 2.05, *p* = 0.244).

Among all 133 cases, including both true lesions and transient non-mass ABUS findings, comparative clinical and ABUS findings are summarized in Table 4. Larger lesion size, presence of microcalcification-like echogenic foci, ductal change, cystic change, and volume change were associated with true lesions, whereas the presence of posterior shadowing, more than half involvement of FGT, and heterogeneous background echotexture were associated with transient non-mass ABUS findings.

**Figure 4:**
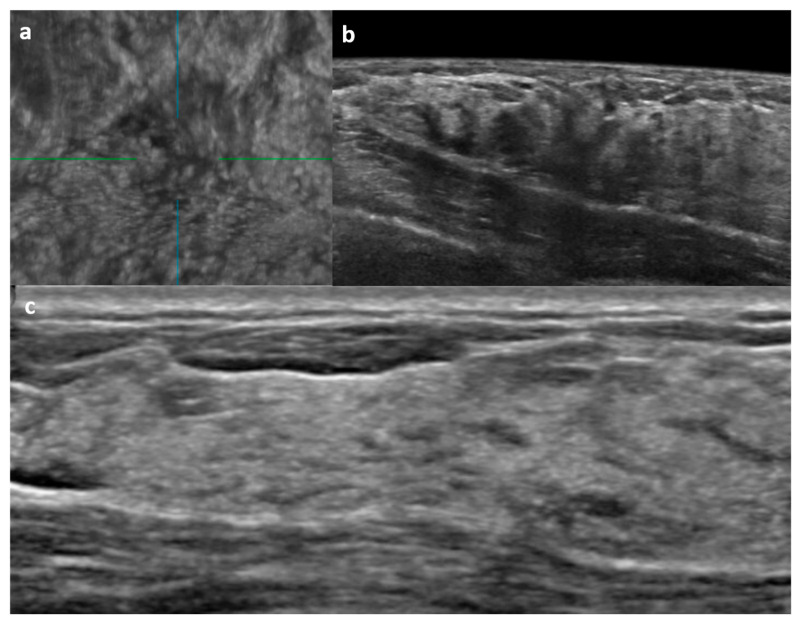
A 54-year-old woman diagnosed with a transient non-mass ABUS finding due to an inadequate compression artifact. (**a**,**b**) The axial and coronal planes of ABUS show a non-mass lesion with posterior shadowing, ≥50% FGT involvement, and no volume change in the upper outer quadrant of the right breast. (**c**) Second-look ultrasound performed subsequently in the same region does not clearly demonstrate a corresponding focal lesion.

Among all 133 cases, multivariable analysis was performed to identify imaging features associated with true lesions rather than transient non-mass ABUS findings (Table 5). Non-heterogeneous background echotexture (OR = 0.07; 95% CI, 0.02–0.33; *p* = 0.001), lack of posterior shadowing (OR = 0.14; 95% CI, 0.03–0.60; *p* = 0.008), and volume change (OR = 15.79; 95% CI, 1.47–170.12; *p* = 0.023) were associated with true lesions rather than transient non-mass ABUS findings (Figure 4).

## 4. Discussion

In this study, 22.1% (15 of 68) of true non-mass lesions detected on ABUS were malignant. Among the malignant non-mass lesions, 53.3% (8 of 15) were invasive cancers. The incidence of invasive cancers aligns with findings reported in previous studies, ranging from 40% to 74% [14,20,21,22]. The presence of invasive cancers among malignant non-mass lesions suggests that careful interpretation of non-mass lesions on ABUS is important. Although several ABUS features were associated with malignancy in univariable analysis, segmental distribution and coronal architectural distortion remained associated with malignancy in the exploratory multivariable analysis. These findings are consistent with previous studies [14,17,22]. Segmental distribution resembles ductal hypoechoic areas or ductal non-mass lesion patterns in the studies by Ko et al. and Uematsu et al. [18,20], which were defined as lesions showing a parallel orientation of multiple duct-like hypoechoic structures towards the nipple area. Ductal hypoechoic areas are indications for tissue confirmation [18,23]. Corresponding to linear or segmental non-mass enhancement on magnetic resonance imaging, which is more likely to indicate ductal malignancy, segmental non-mass lesions have been reported to be associated with DCIS on US [24]. Of the 15 malignant non-mass lesions, nine (60.0%) had a segmental distribution. Among these nine lesions, seven (77.8%) were either pure DCIS or had DCIS components. These findings may also be interpreted in the context of MRI, where segmental or linear non-mass enhancement patterns are known to be associated with ductal malignancy, particularly DCIS [25]. In this regard, segmental distribution on ABUS may reflect a similar underlying pathophysiologic process. According to Zhang et al., ABUS (61.4%) could detect non-mass lesions with architectural distortion better than mammography (38.6%) [17]. The coronal plane is a unique feature of ABUS and has been reported to provide additional visualization of structural abnormalities, sometimes referred to as the “surgical view” [4,17,26]. In this study, there was no significant difference in benign non-mass lesions between architectural distortion on the axial and coronal planes (three lesions, 5.7% versus two lesions, 3.8%). However, architectural distortion was more frequently seen on the coronal plane in malignant non-mass lesions (three lesions, 20.0% vs. 10 lesions, 66.7%). A recent study by Mun et al. [12] reported that ABUS tends to assign lower BI-RADS categories than HHUS for small (≤1 cm) breast cancers while still maintaining suspicion for malignancy. From this perspective, non-mass lesions on ABUS showing segmental distribution or architectural distortion on the coronal plane may be associated with malignancy. Nevertheless, because 2 of the 15 malignant lesions (13.3%) showed neither segmental distribution nor architectural distortion on the coronal plane, the absence of these findings does not exclude malignancy. Therefore, segmental distribution and coronal-plane architectural distortion should be interpreted as exploratory imaging features associated with malignancy rather than diagnostic criteria, particularly given the small number of malignant lesions.

Many previous studies have reported an association between microcalcifications in non-mass lesions and malignancies [14,18,20,22,27,28]. In our study, microcalcification-like echogenic foci were observed in 7.5% and 66.7% of the benign and malignant groups, respectively, in the comparative analysis using Fisher’s exact test (*p* < 0.001). Although microcalcification-like echogenic foci were associated with malignancy in univariable analysis, this association did not reach statistical significance in the multivariable analysis, possibly reflecting the limited number of malignant lesions (*n* = 15). Contrary to recent results [14], the presence of posterior shadowing was not significantly associated with malignancy in our study (OR = 2.05, *p* = 0.244). This finding has been debated in several previous studies. Zhang et al. showed no statistical significance of posterior shadowing in malignant non-mass lesions [28]. In conventional HHUS, posterior shadowing is often considered a suspicious feature associated with desmoplastic reaction in malignant lesions. However, prior studies have suggested that posterior shadowing in non-mass lesions may also reflect a spectrum of pathological changes, ranging from benign fibrosis or scarring to malignant processes [18,20,29,30]. In this context, posterior shadowing on ABUS may be influenced by differences in imaging physics and acquisition techniques. Unlike HHUS, ABUS involves automated scanning using a wide-format transducer and standardized compression, which may result in uneven pressure distribution across the breast. In addition, non-perpendicular incidence of the ultrasound beam and limited probe adjustability may lead to increased attenuation artifacts. These technical factors may contribute to artifactual posterior shadowing (pseudo-shadowing), which does not necessarily reflect true tissue characteristics but rather imaging-related artifacts inherent to ABUS.

To our knowledge, few studies have explored imaging features that may help distinguish true non-mass lesions from transient non-mass ABUS findings. In the multivariable analysis, non-heterogeneous background echotexture, lack of posterior shadowing, and volume change were associated with true lesions rather than transient non-mass ABUS findings. According to Kim et al., ABUS shows a tendency toward a more heterogeneous background echotexture than HHUS, especially in premenopausal women [31]. ABUS can cause more posterior shadowing than conventional US because it may not be possible to properly compress the probe, and reflection at an acute angle to the US beam is less flattened by the transducer [32]. ABUS also has a wide transducer; the presence of a small heterogeneous area may lead to the categorization of heterogeneous background echotexture [5] and the evaluation of the presence of non-mass lesions. In our cohort of 133 women (mean age, 50.75 years; range, 39–62), 60 were perimenopausal. Heterogeneous background echotexture may therefore mimic non-mass lesions on ABUS.

Posterior shadowing on ABUS may reflect artifacts or transient non-mass ABUS findings; therefore, the absence of posterior shadowing was associated with true lesions in our cohort. Consistent with the ACR BI-RADS v2025 update, according to the Japan Society of Ultrasonics in Medicine and the Japan Association of Breast and Thyroid Sonology guidelines, and the recent Society of Breast Imaging symposium [33,34], a mass is defined as a space-occupying lump, whereas a non-mass lesion is defined as a discrete finding distinctly different from normal tissue, without margination or the specific shape of a mass on US. According to this definition, non-mass lesions do not meet the definition of a mass but still occupy a certain amount of space, which may partly explain the observed association with volume change. Skip artifact was significant in the univariable analysis, but not included in the multivariable model because it represents a technical artifact rather than a lesion-related imaging feature.

Our study had several limitations. First, this was a single reader retrospective study performed at a single institution using a single-model ABUS system, which may limit external generalization. Second, the number of malignant cases in our cohort was relatively small (*n* = 15), which represents a key limitation of this study. This result may be due to the nature of our institution and the time-consuming learning curve of ABUS interpretation. In screening US, non-mass lesions requiring needle biopsies are uncommon [35,36]. This limited sample size may reduce statistical power, contribute to wide confidence intervals, and increase the risk of overfitting in the multivariable model, particularly when multiple imaging features are considered. Furthermore, some imaging features previously reported to be associated with malignancy, such as microcalcifications, were not retained in the final model. This may be due to insufficient statistical power rather than definitive evidence of no association. Therefore, our findings should be interpreted with caution and considered exploratory in nature. In addition, the definition of volume change using a 10% threshold was not based on previously validated criteria and was assessed visually in a semi-quantitative manner rather than by automated volumetric measurement. This approach was intended to provide a practical distinction between true lesions and background variation. Several imaging features, including background echotexture, coronal architectural distortion, skip artifact, and volume change, may be observer-dependent. In this study, all image analyses were performed by a single reader, and interobserver agreement was not assessed. Therefore, the reproducibility of these findings remains uncertain and warrants further validation in future studies including multiple readers. Third, by restricting the analysis to BI-RADS categories 0, 4, and 5, our study population was enriched for more suspicious lesions, which may have influenced the observed malignancy rate and the apparent performance of individual imaging features. Therefore, these results should not be generalized to all ABUS-detected non-mass lesions, particularly those assessed as probably benign. In addition, because the classification of some benign lesions and transient non-mass ABUS findings relied on second-look or follow-up ultrasound rather than pathologic confirmation, a non-uniform reference standard was used. This may have introduced verification bias and should be considered when interpreting our findings. Fourth, related to this non-uniform reference standard, two of the 53 benign lesions were classified based on follow-up stability alone. A follow-up period of 12 months may be relatively short for subtle non-mass lesions or low-grade malignancies, and longer follow-up of 24 months or more would provide a stronger reference standard for benignity. Fifth, systematic multimodality correlation with mammography, DBT, and MRI was not performed for the entire cohort. In a descriptive post hoc review, available mammograms were reviewed for potential mammographic correlates only for the malignant lesions, whereas benign lesions and transient non-mass ABUS findings were not evaluated for mammographic correlation. Because microcalcifications are primarily mammographic descriptors, and segmental distribution and architectural distortion may have important mammographic or DBT correlates, the lack of systematic multimodality correlation limits the ability to contextualize these ABUS findings. Therefore, the ABUS findings in this study should be interpreted with caution, and the mammographic correlation described in the descriptive post hoc review should be regarded as supplemental descriptive information. Finally, this study evaluated imaging associations but did not assess screening outcomes, biopsy yield, recall reduction, or management impact. Screening-related clinical factors, such as family history, comorbidities, and menstrual factors, were not systematically collected; therefore, their relationship with the occurrence of non-mass lesions or screening selection could not be evaluated. The practical implications of these findings require further validation.

In conclusion, segmental distribution and coronal architectural distortion were associated with malignancy on ABUS in this exploratory study. Lack of posterior shadowing and the presence of volume change were associated with true lesions rather than transient non-mass ABUS findings, while heterogeneous background echotexture was associated with transient non-mass ABUS findings. These ABUS findings may contribute to the characterization of non-mass lesions. However, given the small number of malignant lesions, the single-reader assessment, and the non-uniform reference standard, these findings should be interpreted with caution. Further validation in larger, multi-reader studies using a uniform reference standard is warranted.

## Figures and Tables

**Table 1:** Reference standards for classification of true lesions and transient non-mass ABUS findings.

Variables	True Lesions (*n* = 68)		Transient Non-Mass ABUS Findings (*n* = 65)
	Benign (*n* = 53)	Malignancy (*n* = 15)	
Second look US ^1^	BI-RADS category 2 or 3		Without a corresponding lesion on second look US (fat lobule, parenchyma, shadowing by Cooper’s ligament)
Pathologic outcome ^2^	Not defined as malignancy	Invasive ductal carcinoma Invasive lobular carcinoma Ductal carcinoma in situ	
Follow up US	Stable lesions for at least 12 months.		Disappeared Definite shadowing artifact

BI-RADS, Breast Imaging Reporting and Data System; US, ultrasound. ^1^ Second look US within 2 weeks when initial BI-RADS category was 0. ^2^ Pathologic outcomes from US-guided core needle biopsy with surgical pathology incorporated when available.

**Table 2:** Comparison of automated breast ultrasound findings between benign and malignant true non-mass lesions (*n* = 68).

Variables	Total (*n* = 68)	Final Pathology		*p*-Value
		Benign (*n* = 53)	Malignancy (*n* = 15)	
Age (years)	51.72 ± 10.96	51.72 ± 10.66	51.73 ± 12.34	0.882 ^1^
Lesion size (mm)	20.84 ± 12.10	19.70 ± 11.93	24.87 ± 12.22	0.076 ^2^
Internal echogenicity				0.057 ^4^
Isoechogenicity	23 (33.8%)	21 (39.6%)	2 (13.3%)	
Hypoechogenicity	45 (66.2%)	32 (60.4%)	13 (86.7%)	
Distribution				<0.001 ^3^
Non-segmental	57 (83.8%)	51 (96.2%)	6 (40.0%)	
Segmental	11 (16.2%)	2 (3.8%)	9 (60.0%)	
Presence of microcalcification-like echogenic foci				<0.001 ^3^
No	54 (79.4%)	49 (92.5%)	5 (33.3%)	
Yes	14 (20.6%)	4 (7.5%)	10 (66.7%)	
Posterior shadowing				0.329 ^3^
No	49 (72.1%)	40 (75.5%)	9 (60.0%)	
Yes	19 (27.9%)	13 (24.5%)	6 (40.0%)	
Ductal change				0.001 ^3^
No	51 (75.0%)	45 (84.9%)	6 (40.0%)	
Yes	17 (25.0%)	8 (15.1%)	9 (60.0%)	
Cystic change				0.102 ^3^
No	58 (85.3%)	43 (81.1%)	15 (100.0%)	
Yes	10 (14.7%)	10 (18.9%)	0 (0.0%)	
Architectural distortion on axial plane				0.116 ^3^
No	62 (91.2%)	50 (94.3%)	12 (80.0%)	
Yes	6 (8.8%)	3 (5.7%)	3 (20.0%)	
Architectural distortion on coronal plane				<0.001 ^3^
No	56 (82.4%)	51 (96.2%)	5 (33.3%)	
Yes	12 (17.6%)	2 (3.8%)	10 (66.7%)	
Skip artifact on coronal plane				0.034 ^3^
No	52 (76.5%)	44 (83.0%)	8 (53.3%)	
Yes	16 (23.5%)	9 (17.0%)	7 (46.7%)	
Location				0.128 ^4^
Neither	23 (33.8%)	15 (28.3%)	8 (53.3%)	
Periareola	14 (20.6%)	13 (24.5%)	1 (6.7%)	
Periphery	31 (45.6%)	25 (47.2%)	6 (40.0%)	
Location				0.070 ^4^
Periareola/periphery	45 (66.2%)	38 (71.7%)	7 (46.7%)	
Neither	23 (33.8%)	15 (28.3%)	8 (53.3%)	
Involvement degree of fibroglandular tissue				0.003 ^4^
Whole involvement	12 (17.6%)	5 (9.4%)	7 (46.7%)	
Less than half	33 (48.5%)	29 (54.7%)	4 (26.7%)	
More than half	23 (33.8%)	19 (35.8%)	4 (26.7%)	
Involvement degree of fibroglandular tissue				0.055 ^4^
More than half/whole involvement	35 (51.5%)	24 (45.3%)	11 (73.3%)	
Less than half	33 (48.5%)	29 (54.7%)	4 (26.7%)	
Volume change				0.491 ^3^
No	54 (79.4%)	43 (81.1%)	11 (73.3%)	
Yes	14 (20.6%)	10 (18.9%)	4 (26.7%)	
Background echotexture				0.353 ^3^
Homogeneous	46 (67.6%)	34 (64.2%)	12 (80.0%)	
Heterogeneous	22 (32.4%)	19 (35.8%)	3 (20.0%)	

BI-RADS, Breast Imaging Reporting and Data System. ^1^ independent *t*-test, ^2^ Mann–Whitney U test, ^3^ Fisher’s exact test, ^4^ Pearson chi-square test.

**Table 3:** Multivariable logistic regression analysis of automated breast ultrasound findings associated with malignancy in true non-mass lesions (*n* = 68).

Variables	Univariable Analysis		Multivariable Analysis	
	Odds Ratio	*p*-Value	Adjusted Odds Ratio *	*p*-Value
Distribution				
Non-segmental	1			
Segmental	38.25	<0.001	40.42 (4.45, 367.27)	0.001
Presence of microcalcification-like echogenic foci				
No	1			
Yes	24.50	<0.001	NA	
Posterior shadowing				
No	1			
Yes	2.05	0.244	NA	
Ductal change				
No	1			
Yes	8.44	0.001	NA	
Architectural distortion on axial plane				
No	1			
Yes	4.17	0.104	NA	
Architectural distortion on coronal plane				
No	1			
Yes	51.00	<0.001	53.59 (6.26, 458.90)	<0.001
Skip artifact on coronal plane				
No	1			
Yes	4.28	0.022	NA	
Involvement degree of fibroglandular tissue				
Whole involvement	1			
Less than half	0.10	0.003	NA	
More than half	0.15	0.018	NA	

* Data in parentheses are 95% confidence intervals; NA, not included in multivariable analysis due to variable selection criteria and limited number of events.

**Table 4:** Comparison of automated breast ultrasound findings between transient non-mass ABUS findings and true lesions in all cases (*n* = 133).

Variables	Total (*n* = 133)	Final Diagnosis		*p*-Value
		Transient Non-Mass ABUS Findings (*n* = 65)	True (*n* = 68)	
Age (years)	50.75 ± 11.36	49.74 ± 11.77	51.72 ± 10.96	0.685 ^1^
Lesion size (mm)	18.77 ± 10.42	16.62 ± 7.83	20.84 ± 12.10	0.042 ^1^
Internal echogenicity				0.073 ^2^
Isoechogenicity	36 (27.1%)	13 (20.0%)	23 (33.8%)	
Hypoechogenicity	97 (72.9%)	52 (80.0%)	45 (66.2%)	
Distribution				0.133 ^2^
Non-segmental	117 (88.0%)	60 (92.3%)	57 (83.8%)	
Segmental	16 (12.0%)	5 (7.7%)	11 (16.2%)	
Presence of microcalcification-like echogenic foci				0.001 ^2^
No	118 (88.7%)	64 (98.5%)	54 (79.4%)	
Yes	15 (11.3%)	1 (1.5%)	14 (20.6%)	
Posterior shadowing				0.001 ^2^
No	77 (57.9%)	28 (43.1%)	49 (72.1%)	
Yes	56 (42.1%)	37 (56.9%)	19 (27.9%)	
Ductal change				<0.001 ^2^
No	115 (86.5%)	64 (98.5%)	51 (75.0%)	
Yes	18 (13.5%)	1 (1.5%)	17 (25.0%)	
Cystic change				0.019 ^2^
No	121 (91.0%)	63 (96.9%)	58 (85.3%)	
Yes	12 (9.0%)	2 (3.1%)	10 (14.7%)	
Architectural distortion on axial plane				0.513 ^2^
No	119 (89.5%)	57 (87.7%)	62 (91.2%)	
Yes	14 (10.5%)	8 (12.3%)	6 (8.8%)	
Architectural distortion on coronal plane				0.156 ^2^
No	115 (86.5%)	59 (90.8%)	56 (82.4%)	
Yes	18 (13.5%)	6 (9.2%)	12 (17.6%)	
Skip artifact on coronal plane				0.474 ^2^
No	105 (78.9%)	53 (81.5%)	52 (76.5%)	
Yes	28 (21.1%)	12 (18.5%)	16 (23.5%)	
Location				0.265 ^2^
Neither	37 (27.8%)	14 (21.5%)	23 (33.8%)	
Periareola	28 (21.1%)	14 (21.5%)	14 (20.6%)	
Periphery	68 (51.1%)	37 (56.9%)	31 (45.6%)	
Location				0.114 ^2^
Periareola/periphery	96 (72.2%)	51 (78.5%)	45 (66.2%)	
Neither	37 (27.8%)	14 (21.5%)	23 (33.8%)	
Involvement degree of fibroglandular tissue				0.027 ^2^
Whole involvement	20 (15.0%)	8 (12.3%)	12 (17.6%)	
Less than half	53 (39.8%)	20 (30.8%)	33 (48.5%)	
More than half	60 (45.1%)	37 (56.9%)	23 (33.8%)	
Involvement degree of fibroglandular tissue				0.037 ^2^
More than half/whole involvement	80 (60.2%)	45 (69.2%)	35 (51.5%)	
Less than half	53 (39.8%)	20 (30.8%)	33 (48.5%)	
Volume change				0.002 ^2^
No	117 (88.0%)	63 (96.9%)	54 (79.4%)	
Yes	16 (12.0%)	2 (3.1%)	14 (20.6%)	
Background echotexture				<0.001 ^2^
Homogeneous	64 (48.1%)	18 (27.7%)	46 (67.6%)	
Heterogeneous	69 (51.9%)	47 (72.3%)	22 (32.4%)	

BI-RADS, Breast Imaging Reporting and Data System. ^1^ Mann-Whitney U test, ^2^ Pearson chi-square test.

**Table 5:** Multivariable logistic regression analysis of automated breast ultrasound findings for differentiating true non-mass lesions from transient non-mass ABUS findings in all cases (*n* = 133).

Variable	Univariable Analysis		Multivariable Analysis	
	Odds Ratio	*p*-Value	Adjusted Odds Ratio *	*p*-Value
Size (mm)	1.05	0.025	NA	
Presence of microcalcification-like echogenic foci				
No	1			
Yes	16.59	0.008	NA	
Posterior shadowing				
No	1			
Yes	0.29	0.001	0.14 (0.03, 0.60)	0.008
Ductal change				
No	1			
Yes	21.33	0.003	NA	
Cystic change				
No	1			
Yes	5.43	0.033	5.90 (0.69, 50.84)	0.106
Involvement degree of fibroglandular tissue				
Whole involvement	1			
Less than half	1.10	0.859	NA	
More than half	0.41	0.095	NA	
Involvement degree of fibroglandular tissue				
More than half/whole involvement	1			
Less than half	2.12	0.038	NA	
Volume change				
No	1			
Yes	8.17	0.007	15.79 (1.47, 170.12)	0.023
Background echotexture				
Homogeneous	1			
Heterogeneous	0.18	<0.001	0.07 (0.02, 0.33)	0.001

* Data in parentheses are 95% confidence intervals; NA, not included in multivariable analysis due to variable selection criteria and limited number of events.

## Data Availability

The datasets used and/or analyzed during the current study are available from the corresponding author upon reasonable request.
